# Protective effect of *Panax ginseng* on AlCl₃-induced liver, kidney, and testis toxicity in male albino rats: biochemical, histopathological, and molecular study

**DOI:** 10.3389/fvets.2026.1925382

**Published:** 2026-09-16

**Authors:** Fatimah A. Al-Saeed, Sayed Soliman Abdel Ghfar, Montaser Elsayed Ali

**Affiliations:** 1 Department of Biology, College of Science, King Khalid University, Abha, Saudi Arabia; 2 Department of Animal Productions, Faculty of Agriculture, Al-Azhar University, Cairo, Egypt; 3 Department of Animal Productions, Faculty of Agriculture, Al-Azhar University, Assiut, Egypt

**Keywords:** aluminum chloride (AlCl3), hepatotoxicity, nephrotoxicity, *Panax ginseng*, SIRT1, testicular toxicity

## Abstract

Aluminum chloride (AlCl₃) is a ubiquitous environmental toxicant that induces multi-organ toxicity, primarily via oxidative stress and inflammatory mechanism cascades. *Panax ginseng*, renowned for its pharmacologically active ginsenosides, exhibited potent antioxidant and anti-inflammatory activities. Accordingly, this study aimed to evaluate the protective effects of *Panax ginseng* extract against AlCl₃-induced liver, kidneys, and testes damage, with a specific focus on the expression of NAD-dependent protein deacetylase sirtuin-1 (SIRT1) as a potential mediator. Forty-five male albino rats (*Rattus norvegicus*), weighing 155.80 g ± 0.52 g at 82 days of age, were randomly assigned into three experimental groups (n = 15 in each): control group (CG); AlCl₃-treated group (AlCl₃ group), which received 300 mg/kg body weight/day of AlCl₃·6H₂O via oral gavage daily for 28 days; and co-treatment group (AlCl₃/P-G), 300 mg/kg body weight/day of AlCl₃·6H₂O concurrently with 250 mg/kg/BW *Panax ginseng* for 28 days. This study integrated three analytical pillars: biochemical profiling of serum lipids and liver/kidney function markers alongside SIRT1 protein assessment using ELISA; histopathological evaluation of hepatic, renal, and testicular tissues utilizing H&E staining; and qPCR analysis of SIRT1 mRNA expression. The AlCl₃/P-G group demonstrated weight improvement on days 21 and 28 of treatment compared to AlCl₃ and CG groups. It exhibited significantly lower lipid profile values than AlCl₃ (*p* < 0.01). *Panax ginseng* treatment in this group led to improved creatinine, urea, ALT, and AST levels compared to AlCl₃, along with a significant increase in serum SIRT1 protein concentration and hepatic SIRT1 mRNA expression (0.70-fold of CG levels) compared AlCl₃ (0.50-fold). The treatment also partially restored renal architecture with compact glomeruli and reduced tubular degeneration, as well as hepatic architecture showing reduced swelling and less congestion. Testis sections in the AlCl₃/P-G group showed recovery in epithelial organization and increased spermatogenic activity; however, causal mechanisms remain to be established. In conclusion, *Panax ginseng* may have protective effect on AlCl₃-induced liver, kidney, and testicular toxicity.

## 1 Introduction

Aluminum chloride (AlCl₃) is ubiquitous environmental compound extensively utilized in water treatment, food processing, packaging materials, cosmetics, and various industrial applications. According to the European Food Safety Authority (EFSA), the tolerable weekly intake for elemental aluminum has been established at 1 mg/kg body weight per week, which serves as a reference value for dietary exposure assessment (1). While dietary aluminum intake is minimal, higher doses of AlCl₃ used in experimental studies are important for exploring toxicological mechanisms. Aluminum is primarily eliminated renally, whereas only a minor fraction is absorbed enterally (2, 3). Importantly, chronic aluminum exposure has been associated with neurodegenerative disorders, cardiovascular diseases, hepatorenal dysfunction, and reproductive toxicity in epidemiological and experimental studies (4, 5); however, extrapolating finding from animal models to humans requires caution. The 300 mg/kg of AlCl₃ dose applied in this study constituted a significant toxic challenge, surpassing typical human exposure levels, and was intended to clarify toxicity mechanisms and evaluate treatments.

Aluminum toxicity leads to oxidative stress and inflammation, inhibiting the SIRT1-Keap1/Nrf2 pathway, disrupting antioxidant defenses, and promoting apoptosis (6). In the kidneys, it causes nephrotoxicity marked by oxidative damage, inflammation, and endoplasmic reticulum stress, resulting in functional impairments and structural damage. Elevated blood urea and creatinine levels are indicative of kidney dysfunction, along with tissue degeneration and necrosis (7).

Many studies have shown deterioration of liver enzymes, fibrosis of liver cells, apoptosis, oxidative stress, and metabolic disturbance (8, 9). Exposure to aluminum has been linked to testicular dysfunction, including abnormal testosterone levels, deterioration of semen quality, pathological tissue lesions in the seminiferous tubules, degeneration, and atrophy (10).

*Panax ginseng* and its active components, ginsenosides, exhibit pharmacological effects such as antioxidant, anti-inflammatory, and immune-boosting properties, particularly through enhanced lymphocyte proliferation and white blood cell activation in rodents (11–13). Key constituents include triterpenoid saponins and polysaccharides, with ginsenosides Rb1, Rb2, Rc, Rg1, and Re constituting over 80% of the content, which enhances immune function and provides antioxidant benefits (14). Polysaccharides additionally help downregulate inflammatory responses by reducing pro-inflammatory cytokine expression (15). Furthermore, *Panax ginseng* exhibits protective and protective effects against AlCl₃ toxicity, manifested in the modulation of antioxidants and the reduction of inflammation and programmed cell death (16). Additionally, ginsenosides enhance hepatic glutathione levels and enzyme activities, leading to significant antioxidant and anti-inflammatory effects. They reduce lipid peroxidation, lower inflammatory cytokines, thereby protecting against liver inflammation (17). Moreover, ginsenosides reduce damage, inflammation, and fibrosis by lowering urinary protein and serum levels of creatinine and urea nitrogen, as well as diminishing glomerular fibrosis in diabetes mellitus mouse models (18, 19).

NAD-dependent protein deacetylase sirtuin-1 (SIRT1) regulates oxidative stress, inflammation, energy metabolism, and mitochondrial function (20). It enhances antioxidant effects by deacetylating targets like PGC1α and Nrf2, promoting mitochondrial biogenesis. Aluminum exposure inhibits SIRT1 (21), while activating SIRT1 pharmacologically can mitigate aluminum toxicity. Ginsenosides, particularly Rg1 from *Panax ginseng*, have been reported to activate SIRT1 and boost its activity in previous studies (22).

Despite known antioxidant properties of ginsenosides and their activation of SIRT1, the protective potential of *Panax ginseng* against AlCl₃-induced multi-organ toxicity is under-researched. Given the widespread presence of AlCl₃ and its impact on organ functions, there is a pressing need for effective treatment options. The study was based on the hypothesis that *Panax ginseng*, through its pharmacologically active ginsenosides, ameliorates AlCl₃-induced multi-organ toxicity by modulating SIRT1 expression and/or potentially its activity, reducing lipid peroxidation, and preserving the structural and functional integrity of hepatic, renal, and testicular tissues. Therefore, this study aimed to evaluate the protective effects of *Panax ginseng* extract against AlCl₃-induced toxicity on the liver, kidneys, and testes through assessment of histopathological alterations, biochemical parameters, and SIRT1 gene expression.

## 2 Materials and methods

### 2.1 Aluminum chloride (AlCl₃) exposure

The presented study applied a dosage of 300 mg/kg body weight of aluminum chloride hexahydrate (AlCl₃·6H₂O) in a 28-day oral toxicity assessment, following the methodology described by Lim et al. (23). The AlCl₃·6H₂O was obtained from Al-Gomhouria Company, Cairo, Egypt. A 50 mg/mL stock solution was prepared by dissolving 5.0 g of AlCl₃·6H₂O in distilled water, and the volume was adjusted to 100 mL in a volumetric flask. Complete dissolution was ensured to guarantee stability, given the susceptibility of aluminum chloride hydrolysis. Oral administration was performed via gavage using a stainless-steel feeding tube attached to a 5-mL syringe. The daily gavage volume for each animal was calculated daily using the following formula: $\text{Gavage Volume}=\frac{\text{Body Weight}\;(g)\;x\;300\;\mathrm{mg}/\mathrm{kg}}{\;1000\;g/\mathrm{kg}\;x\;50\;\mathrm{mg}/\mathrm{mL}}$ . The body weight of each animal was recorded daily prior to administration, and the gavage volume was adjusted based on each animal weight to ensure each animal consistently received the target dose throughout the 28 days treatment period. All doses were administered daily at 10:00 a.m. for the entire during of the study.

### 2.2 Experimental design

#### 2.2.1 Experimental location

The study was conducted at the Animal Production Department, Faculty of Agriculture, Al-Azhar University, Cairo, Egypt, located on the Nile River, 160 km (100 mi) from the Mediterranean Sea and 135 km (80 mi) west of the Red Sea, at latitude 27.18° N and longitude 31.19° E.

#### 2.2.2 Animals, housing and experimental design

The study included forty-five male albino rats (*Rattus norvegicus*), weighing 155.80 g ± 0.52 g at 82 days of age. The animals were obtained from the Animal Production Department, Faculty of Science, Al-Azhar University, Cairo, Egypt. The animals were housed in polypropylene cages (dimensions: 45 × 30 × 20), with maximum density of five rats per cage, under standard laboratory conditions: temperature 22 ± 2 °C, relative humidity 50–60%, and a 12-h light/dark cycle.

The rats were provided with a standard rodent pellet diet and water ad libitum. After a one-week adaptation period, the animals were randomly assigned to three groups (n = 15 per each) using a computer-generated random number sequence (Microsoft Excel, RAND function). The experimental groups were control group (CG); AlCl₃-treated group, which received 300 mg/kg body weight/day of AlCl₃·6H₂O via oral gavage (AlCl₃ group); and co-treatment group, which received 300 mg/kg body weight/day of AlCl₃·6H₂O concurrently 250 mg/kg *Panax ginseng* extract via oral gavage (AlCl₃/P-G). All treatments were administered daily for 28 consecutive days at 8:00 p.m. The sample size for each group was calculated using G*Power, requiring 15 animals per group based on an effect size of 0.40, a significance level of α = 0.05, and a power of 0.80. All animals were included in the assessments as no exclusions were necessary, since none met the criteria of over 20% weight loss, severe neurological symptoms, or inability to access water. The individual rat was considered the experimental unit for all analyses.

A commercial extract of *Panax ginseng* root (Batch No. RS20260122, 10:1 extract ratio) was sourced from Purita Co., Ltd. (China) through Health Care Company (Alexandria, Egypt). The extract was produced using 70% ethanol as the extraction solvent: drying ginseng roots were pulverized and subjected to reflux extraction with 70% ethanol at 60–70 °C for 6 h, following the method described by Kim et al. (24), and the resulting extract was concentrated under reduced pressure using a rotary evaporator. The total ginsenoside content of the specific batch used in this experiment was determined by the manufacturer using HPLC with UV detection and was reported as ≥ 5.0% (w/w) in the Certificate of Analysis (COA; Table 1). The COA also confirmed compliance with physiochemical standards and acceptable limits for heavy metals and total plate count, yeast/mold, and microbial contaminants. The selected dose of 250 mg/kg body weight of *Panax ginseng* extract was according to published literature showing its efficacy and safety in rodent models, notably demonstrated by Lee et al. (25), which highlighted significant hepatoprotective effects in liver disease model. *Panax ginseng* was oral administration directly performed via gavage using a stainless-steel feeding tube attached to a 5-mL syringe without any additional solvents or dispersants at 8:00 p.m. daily for 28 consecutive days.

**Table 1:** Certificate of analysis of *Panax ginseng* extract.

Item	Specification	Result
Product name	Ginseng extract	Ginseng extract
Latin name	*Panax ginseng* C.A. Mey	*Panax ginseng* C.A. Mey
Batch number	RS20260122	RS20260122
Part used	Root	Root
Manufacture date	2026-01-22	2026-01-22
Expiry date	2028-01-21	2028-01-21
Ratio	10:1	Complies
Appearance	Light-yellow powder	Complies
Odor & taste	Characteristic odor and taste	Complies
Loss on drying	≤ 5.00%	3.60%
Ash content	≤ 5.00%	2.63%
Particle size	99% through 80 mesh	Complies
Heavy metals (total)	< 10 ppm	Complies
└ Lead (Pb)	< 3 ppm	Complies
└ Cadmium (Cd)	< 1 ppm	Complies
└ Mercury (Hg)	< 0.1 ppm	Complies
└ Arsenic (As)	< 1 ppm	Complies
Total plate count	≤ 1,000 CFU/g	Complies
Yeast & mold	≤ 100 CFU/g	Complies
*E. coli*	Negative	Negative
*Salmonella*	Negative	Negative

### 2.3 Biochemical analyses

At the termination of the experiment, animals were first anesthetized via intraperitoneal injection of Ketamine (50 mg/kg) and xylazine (5 mg/kg). Deep anesthesia were confirmed by the absence of pedal reflexed. Blood samples were then collicted from the retro-orbital plexus using a capillary tube from all 45 animals. Following blood collicthion, euthanasia was performed via cervical dislocation under death anesthesia. Death was confirmed by assessing the cessation of cardiac and respiratory activity. Blood samples were centrifugation at 3000 × g for 15 min, and the serum was extracted and stored at −20 °C untel biochemical analysis. Serum albumin and total protein were analyzed using commercial Spinreact kits (Spinreact, Girona, Spain), according to the manufactures instructions (26). However, urea, alanine transaminase (ALT), aspartate transaminase (AST), and Creatinine were determined using Biomed-GPT-kinetic kits (27). Hannover, Germany. The serum concentration of total cholesterol (TC), triglycerides (TG), high-density lipoprotein (HDL), and lactate dehydrogenase (LDH) were measured directly using commercial colorimetric kits (spinreact, Girina, Spain) according to the maufacturere instructions (28); however, lactate dehydrogenase (LDH) was calculated using the formula: LDL = TC-HDL-(TG/5) (29). The concentration of NAD-dependent protein deacetylase sirtuin-1 (SIRT1) in the samples was estimated using enzyme-linked immunosorbent assay (ELISA). This study relied on the use of a commercial SIRT1 measurement kit, supplied by Antibodies online, Aachen, Germany: catalog number ABIN6970228.

### 2.4 Histopathological examination

After blood collection, dissection was immediately performed to collect histological specimens. Liver, kidney, and testis were removed from all animals (n = 15 per group), washed with cold phosphate-buffered saline (PBS), and dried. Each organ was divided into two parts: one part was fixed in 10% neutral buffered formalin for histopathological examination, and the second part was stored at −80 °C for gene expression analysis. After 48 h of formalin fixation, tissues were trimmed to no more than 4 mm thickness dehydrated through graded ethanol series (70, 90, and 100%), cleared in xylene, and embedded in paraffin wax. Sections (4–5 μm) were cut using a rotary microtome, stained with hematoxylin and eosin (H&E) following standard protocols, and examined under a light microscope (LABOMED L × 400; Myobone Corporation, Houston, TX, United States; scale bar: 100 μm; magnification ×100 and ×400). Tissue section were quantitative graded (0–4) according to Olcekci and Adiguzel (30), where scores reflected the percentage of histopathological damage: 0 (none), 1 (<25%), 2 (25–50%), 3 (50–75%), and 4 (<75%). For quantitative assessment, three non-overlapping sections per organ were evaluated, capturing ten randomly selected microscopic fields at ×400 magnification to minimize bias. All evaluations were conducted by a blinded pathologist using predefined scoring criteria. The assessment involved histopathological scoring of hepatic, renal, and testicular tissues, focusing on specific conditions: hepatic tissues included hepatocellular vacuolation. The mean score was calculated for each animal, serving as the biological unit in the statistical analysis. A second independent pathologist re-evaluated 20% of randomly selected slides to confirm scoring consistency, as inter-observer reproducibility was not applicable with only one pathologist performing the scoring. For histopathological assessment, three non-overlapping sections from each organ per animal were analyzed. Ten randomly chosen microscopic fields at ×400 magnification were captured using systematic random sampling to prevent observer bias.

### 2.5 Analysis of SIRT1 gene expression by quantitative real-time PCR (qPCR)

The gene expression level of SIRT1 was estimated using real-time quantitative polymerase chain reaction (qPCR). Total RNA was extracted from liver tissue samples using TRIzol™ Reagent (cataloge number: 15596026, Life Technologies, United States) according to the manufacturer’s protocol. RNA purity and concentration were assessed using a NanoDrop™ spectrophotometer (Thermo Fisher Scientifif, United States). RNA integrity was verified by analyzing ribosomal RNA bands on a 1% denaturing agarose gel. One microgram of total RNA was reverse-transcriped into single-stranded cDNA using the QuantiTect™ (Qiagen, United States), following the manufacturer’s instructions, which incloding a gDNA Wipeout Buffer step to eliminate any genomic DNA contamination. The synthesized cDNA samples were stored at −20 °C until future use. Quantitative real-time PCR was performed using a Rotorr-Gen Q system, each reaction mixture contaned 30 ng of cDNA template, 300 nM of each gene-specific primers, and 1x Maxima SYBR Green/Fluorescein qPCR Master Mix in a total volum 20 μL. Primer amplification efficiency was validated using a standard curve generated from a dilution series of pooled cDNA. All primers demonstreated efficiencies within the acceptable range (SIRT1: 97%; GAPDH: 102%), with correlation coefficients (R^2^) exceeding 0.99. Amplification specificity was confirmed by the presence of single peak in the melt-curve analysis for each primer pair. Each sample was analyzed and a no-template control (NTC) was included for primer pair to monitor for potential contamination. Threshold cycle (Cq) values were calculated by Rotor-Gene Q system. GAPDH was employed as the reference (housekeeping) gene for data normalized. Primer sequences for SIRT1 and GAPDH are listed in Table 2. Relative gene expression was calculated using the ^2^-ΔΔ^Ct method, with the CG serving as the calibrator.

**Table 2:** Selected primers for NAD-dependent protein deacetylase sirtuin-1 (*SIRT1*) gene amplification.

Genes	Primer sequences (5′ → 3′)	Reference
SIRT1	5′ – GTCTGTGCCTTCCAGTTGCT-3′ 5′ – CTGCTTGCTGTCCATACCTG-3’	(46)
GAPDH	5’ – ACAGCAACAGGGTGGTGGAC-3′ 5′ – TTTGAGGGTGCAGCGAACTT-3’	

### 2.6 Statistical analysis

The data were statistically analyzed using SPSS for Windows version 25 (SPSS Inc., Chicago, IL, United States). The Shapiro–Wilk test confirmed that the data followed a normal distribution, while Levene’s test was utilized to assess the homogeneity of variances. One-way ANOVA with Tukey’s-hoc test was applied to biochemical parameters, SIRT1 protien concentration, RT-qPCR values. RT-qPCR results are expressed as fold-change (^2^-ΔΔ^Ct) compared to the control group, while all statistical analyses are based on ^Δ^Cq values. However, Body weight (BW) data were analyzed using a repeated-measures general linear model and a two-way ANOVA, considering group and time as fixed effects and addressing their interaction, while including treatment as a fixed factor. The data, reported as mean ± SEM, included 15 biological replicates per group, with statistical significance set at *p* < 0.05 and highly significant at *p* < 0.01. Individual data points are available in the supplementary materials, following the model: Y_ij_ = μ + A_j_ + T_i_ + E_ij_, where Y_ij_ = experimental observation, μ = general mean, A_j_ = effect of Time (Only in BW), T_i_ = groups (_i_ = CG, AlCl₃, and AlCl₃/*P-G*), and _ij_ = experimental error.

## 3 Results

### 3.1 Body weight (BW)

To evaluate changes in BW data, a two-way repeated-measures ANOVA was performed, with group (Control, AlCl₃, and AlCl₃ + *P-G*) and time (Days 0, 7, 14, 21, and 28) as fixed factors, along with their interaction time (Table 3). The statistecal significant defferences were observed in inicial BW among groups (*p* > 0.05). The analysis revealed a significant group × time interaction (*p* < 0.001). Additionally, the main effects of time and group were also significant (*p* < 0.001). At days 7 and 14, BW in both the AlCl₃ and AlCl₃/P-G groups were significant lower than those in the CG (*p* < 0.001), with reduction being more pronouced in the AlCl₃ group. Conversely, at days 21 and 28, the AlCl₃/P-G group exhibited significant in BW compared to the AlCl₃ group; however, it still weights less than the CG at both time.

**Table 3:** Body weight parameter (g) in CG, AlCl_3_, and AlCl_3_/P-G (n = 15 /group, mean ± SEM).

Body weight parameter (g)	CG	AlCl₃	AlCl₃/*P*-G	Overall mean	SEM	95% CI (Lower–Upper)	Sig
Initial	155.90	155.70	153.80	155.80	0.52	153.35–158.25	0.9885
On 7th Day	154.35^a^	144.90^b^	149.20^b^		1.37	147.04–151.93	<0.0127
On 14th Day	167.00^a^	149.30^b^	159.90^b^		2.02	156.29–161.18	<0.0003
On 21st Day	190.00^a^	156.25^c^	165.00^b^		2.95	167.97–172.86	<0.0001
On 28th Day	195.60^a^	165.51^b^	173.71^c^		2.68	175.82–180.71	<0.0001
95% CI (Lower–Upper)	170.68–174.47	152.44–156.23	158.83–162.62				

Tests of between-subjects effects					
Source	Type III sum of squares	df	Mean square	F	Sig.
Time	16192.943	4	4048.236	88.158	<0.0001
Group	8565.870	2	4282.935	93.269	<0.0001
Time * Group	4447.197	8	555.900	12.106	<0.0001

^a, b, c^ = Means with different superscripts in the same row differ significantly. *p* ≤ 0.05; *p* ≤ 0.01; ns = not significant.

### 3.2 Biochemical parameters

Total Protein (g/dl) decreased significantly (*p* < 0.05) in the AlCl₃ group compared to CG and AlCl₃/P-G, while the AlCl₃/P-G group showed significantly (*p* < 0.01) higher values than the AlCl₃ group (Table 4). Moreover, the AlCl₃ and AlCl₃/P-G groups showed a significant (*p* < 0.01) decrease in the albumin levels (g/dl) compared to the CG. Regarding the lipid profile, total cholesterol (TC), triglyceride (Tg), low-density lipoprotein (LDL), and high-density lipoprotein (HDL) increased (*p* < 0.01) in the AlCl₃ group compared to the CG and AlCl₃/P-G groups. Acetate dehydrogenase (LDH), measured as marker of the tissue damage, also increased significantly (*p* < 0.01) the AlCl₃ group compared to the CG and the AlCl₃/P-G. In contrast, the AlCl₃/P-G showed significantly (*p* < 0.01) lower values compared to the AlCl₃ group with levels approaching those of the CG (Table 4). Furthermore, AlCl₃ group exhibited significantly (*p* < 0.01) increases in creatinine, urea, alanine transaminase (ALT), and aspartate aminotransferase (AST), compared to CG. The AlCl₃/*P-G* groups showed a significant (*p* < 0.01) improvement in these parameters compared to the AlCl₃ group (*p* < 0.01).

**Table 4:** Serum biochemical parameters in CG, AlCl_3_, and AlCl_3_/P-G (n = 15 /group, mean ± SEM).

Parameter	CG	AlCl₃	AlCl₃/*P*-G	SEM	Sig
Total protein (g/dl)	8.66^a^	6.27^c^	7.59^b^	0.19	<0.0001
Albumin (g/dl)	5.64^a^	4.51^b^	4.61^b^	0.11	<0.0012
Total cholesterol (TC; mg/dL)	78.83^b^	126.33^a^	77.33^b^	3.58	<0.0001
Triglyceride (Tg; mg/dL)	124.50^c^	295.67^a^	160.83^b^	8.25	<0.0001
Low-density lipoprotein (LDL; mg/dL)	24.67^c^	55.83^a^	43.02^b^	3.25	<0.0001
High-density lipoprotein (HDL; mg/dL)	36.83^c^	76.33^a^	60.83^b^	4.00	<0.0001
lactate dehydrogenase (LDH; U/L)	366.04^c^	687.83^a^	530.17^b^	9.24	<0.0001
Creatinine (mg/dL)	0.78^c^	1.37^a^	0.96^b^	0.05	<0.0001
Urea (mg/dL)	49.76^c^	55.27^a^	34.88^b^	1.73	<0.0030
alanine transaminase (ALT; U/L)	42.49^b^	85.50 ^a^	43.96^b^	1.64	<0.0001
aspartate aminotransferase (AST; U/L)	131.85^c^	361.68^a^	252.76^b^	4.61	<0.0012

^a, b, c^ = Means with different superscripts in the same row differ significantly. *p* ≤ 0.05; *p* ≤ 0.01.

### 3.3 Histopathological findings

Histological examination of the renal sections revealed intact glomerular and tubular architecture in the CG (Figure 1A). The AlCl₃ group exhibited severe pathological disruptions, characterized by distorted glomeruli, overt tubular epithelial degeneration, and conspicuous luminal dilatation (Figure 1B). Conversely, the AlCl₃/P-G group demonstrated a slight, yet non-significant improvement compared to AlCl₃ group, as evidenced by reduced tubular degenerative changes; nevertheless, mild residual abnormalities persisted (Figure 1C). The quantitative histopathological scoring of renal tissues revealed that the AlCl₃/*P-G* group exhibited a slight, yet statistically non-significant (*p* > 0.05), numerical increase in the mean injury scores for glomerular atrophy/collapse, tubular epithelial degeneration, tubular luminal dilatation, and tubulointerstitial inflammation compared to the AlCl₃ group (Table 5). However, H&E-stained liver sections from the CG revealed well-preserved hepatic architecture, with distinct arrangement and intact sinusoidal spaces (Figure 2A). in contrast the AlCl₃ -treated group showed significant histopathological changes such as cytoplasmic vacuolation, ballooning, ballooning, nuclear pyknosishydropic degeneration, loss of regular cordial alignment, and periportal inflammatory cell infiltration (Figure 2B). The AlCl₃/P-G treatment resulted in a partial restoration of normal hepatic architecture, characterized by reduced swelling, fewer vacuolated cells, moderately organized hepatic cords, and less prominent sinusoidal congestion compared to the AlCl₃ treatment (Figure 2C). The quantitative histopathological scoring of hepatic tissues revealed that the AlCl₃/P-G group exhibited a slight, yet statistically non-significant (*p* > 0.05), numerical increase in the mean injury scores for ballooning degeneration, necrosis, and portal/lobular inflammation, with a significant lower in vacuolation compared to the AlCl₃ group (Table 5). In the CG test, testicular sections exhibited normal seminiferous tubules with intact basement membranes, Germinal Epithelium, and well-organized spermatogenic layers (Figure 3A). However, histological analysis of AlCl₃ group revealed significant degenerative changes in the testis, including irregular seminiferous tubules, thinning of the germinal epithelium, a reduction in spermatogenic cell layers, tubule disorganization, and decreased Leydig cell density (Figure 3B). In the AlCl₃/P-G treated group, testis sections showed moderate recovery, with improved epithelial organization of seminiferous tubules, increased spermatogenic activity, reduced degeneration, and better preservation of interstitial Leydig cells (Figure 3C). The quantitative histopathological scoring of testicular tissues revealed that the AlCl₃/P-G group exhibited a statistically higher (*p* < 0.01) in the mean injury scores for seminiferous epithelial disorganization, germ Cell depletion, tubular atrophy, and leydig cell numerical reduction compared to the AlCl₃ group (Table 5). Overall, both the AlCl₃ and AlCl₃/P-G groups demonstrated significantly higher scores (*p* < 0.01) for histopathological scoring of renal, hepatic, and testicular tissues parameters relative to the CG (Table 5).

**Figure 1:**
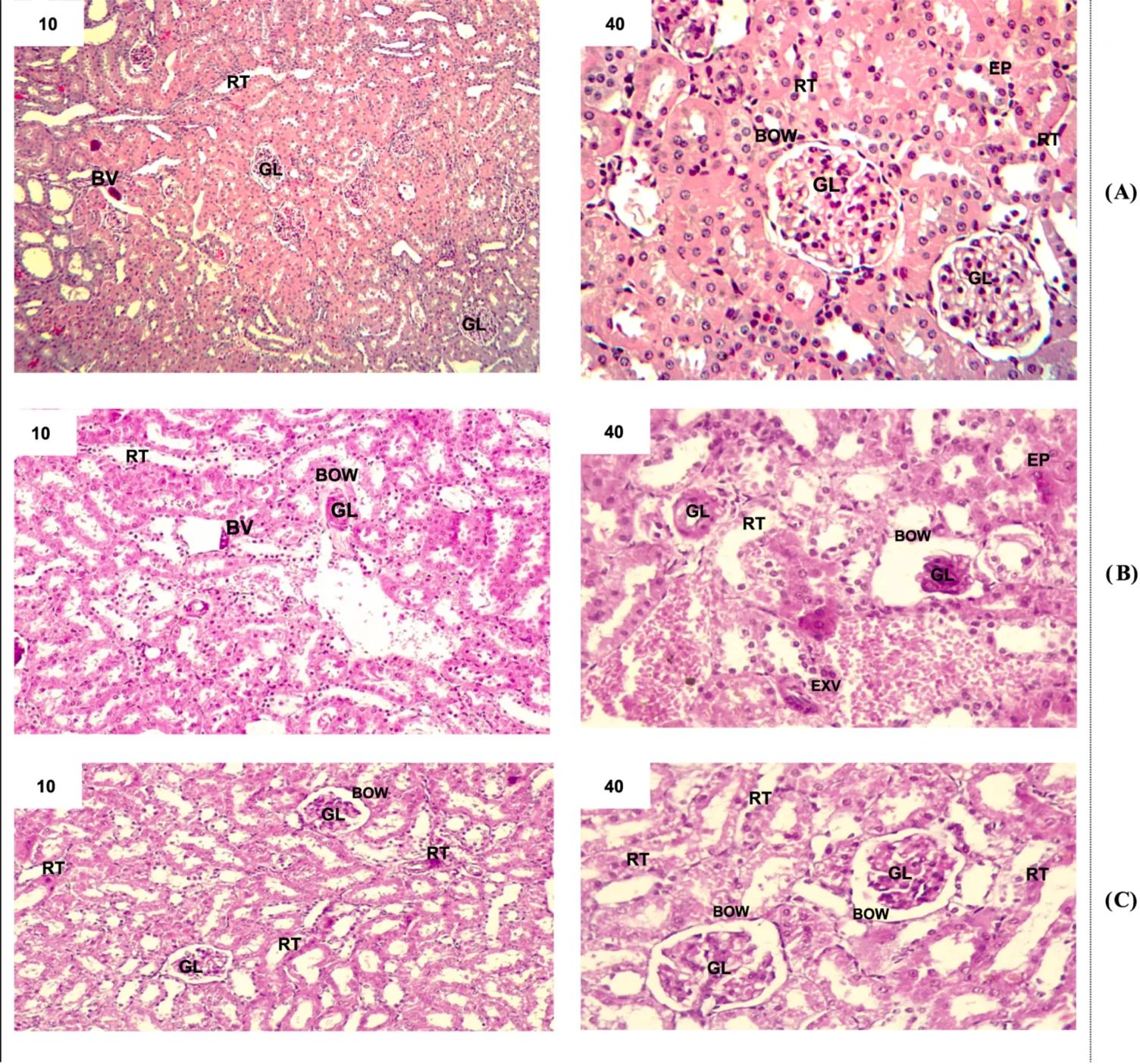
Protective effect of *Panax ginseng* on AlCl-nduced idney in the CG **(A)**, AlCl **(B)**, and AlCl/P-G **(C)**. Glomeruli (GL), Bowman’s capsule (Bow), blood vessels (BV), Renal tubules (T), epithelium (EP), extravasation (EXV).

**Table 5:** Quantitative histopathological scoring of hepatic, renal, and testicular tissues of CG, AlCl_3_, and AlCl_3_/P-G, CG, AlCl_3_, and AlCl_3_/P-G (*n* = 15 /group, mean ± SEM).

Parameter	CG	AlCl₃	AlCl₃/*P-G*	SEM	Sig
Histopathological scoring of renal tissues					
Glomerular atrophy/collapse	0.00^c^	2.40^a^	1.60^a^	0.20	<0.0001
Tubular epithelial degeneration	0.00^b^	1.60^a^	1.30^a^	0.15	<0.0001
Tubular luminal dilatation	0.00^b^	1.70^a^	1.30^a^	0.17	<0.0001
Tubulointerstitial inflammation	0.00^b^	2.50^a^	1.90^a^	0.21	<0.0001
Histopathological scoring of hepatic tissues					
Vacuolation	0.00^c^	2.40^a^	1.70^b^	0.21	<0.0001
Ballooning degeneration	0.00^b^	2.40^a^	2.20^a^	0.22	<0.0001
Necrosis	0.00^b^	2.20^a^	1.80^a^	0.20	<0.0001
Portal/lobular inflammation	0.00^b^	1.90^a^	1.80^a^	0.18	<0.0001
Histopathological scoring of testicular tissues					
Seminiferous epithelial disorganization	0.00^c^	2.20^a^	1.50^b^	0.20	<0.0001
Germ Cell depletion	0.00^c^	2.30^a^	1.20^b^	0.20	<0.0001
Tubular atrophy	0.00^c^	1.60^a^	0.90^b^	0.15	<0.0001
Leydig cell numerical reduction	0.00^c^	2.60^a^	1.20^b^	0.22	<0.0001

^a, b, c^ = Means with different superscripts in the same row differ significantly. *p* ≤ 0.05; *p* ≤ 0.01.

**Figure 2:**
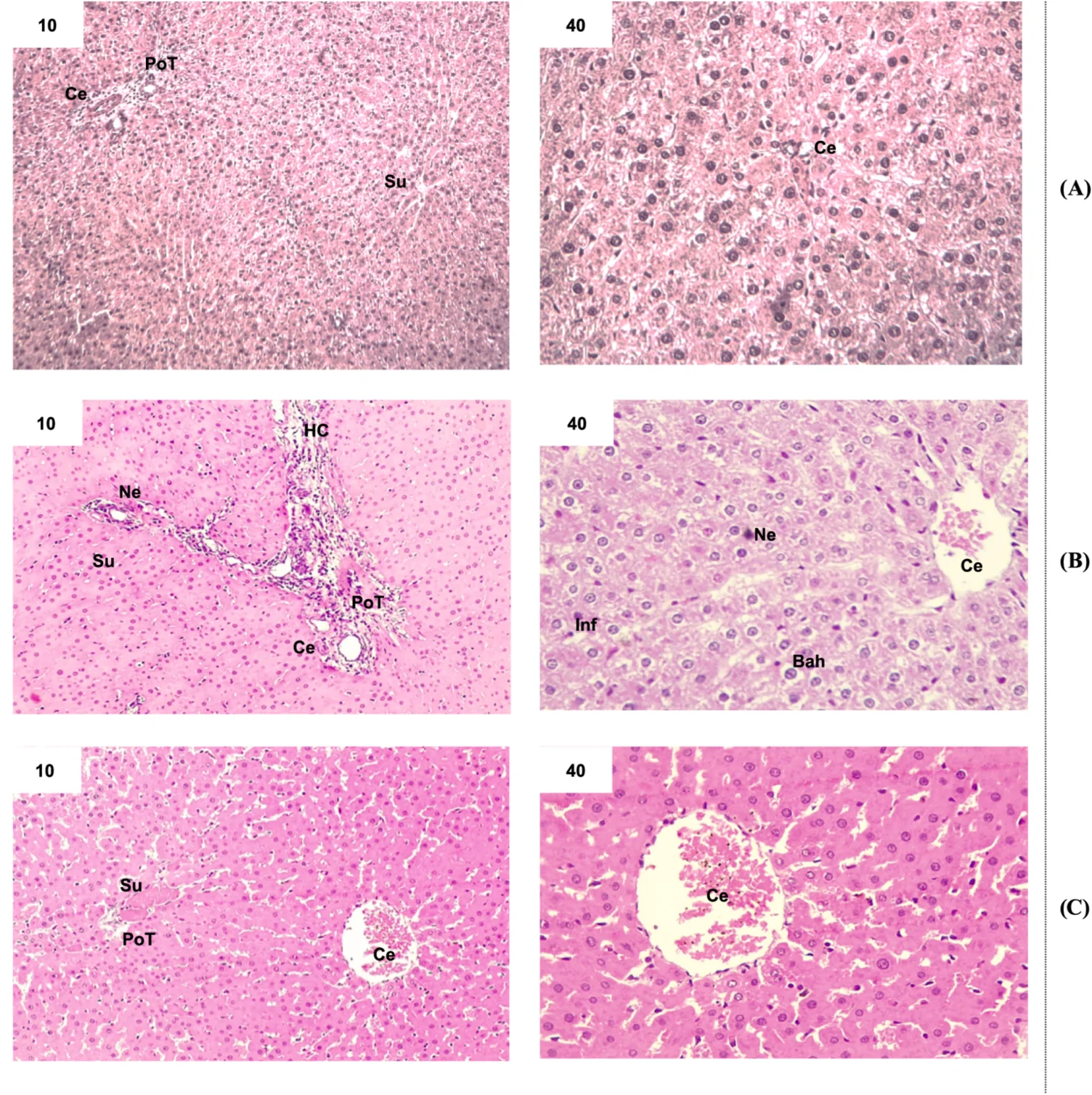
Protective effect of *Panax ginseng* on AlCl-nduced iver in the CG **(A)**, AlCl **(B)**, and AlCl/P-G **(C)**. Central vein (CeV), sinusoids (Su), portal triad (PoT), hepatic cords (HC), allooned hepatocyte (Bah), inflammatory infiltrate (Inf), and ecrosis (Ne). Images are captured by 10X and 40X magnifications.

**Figure 3:**
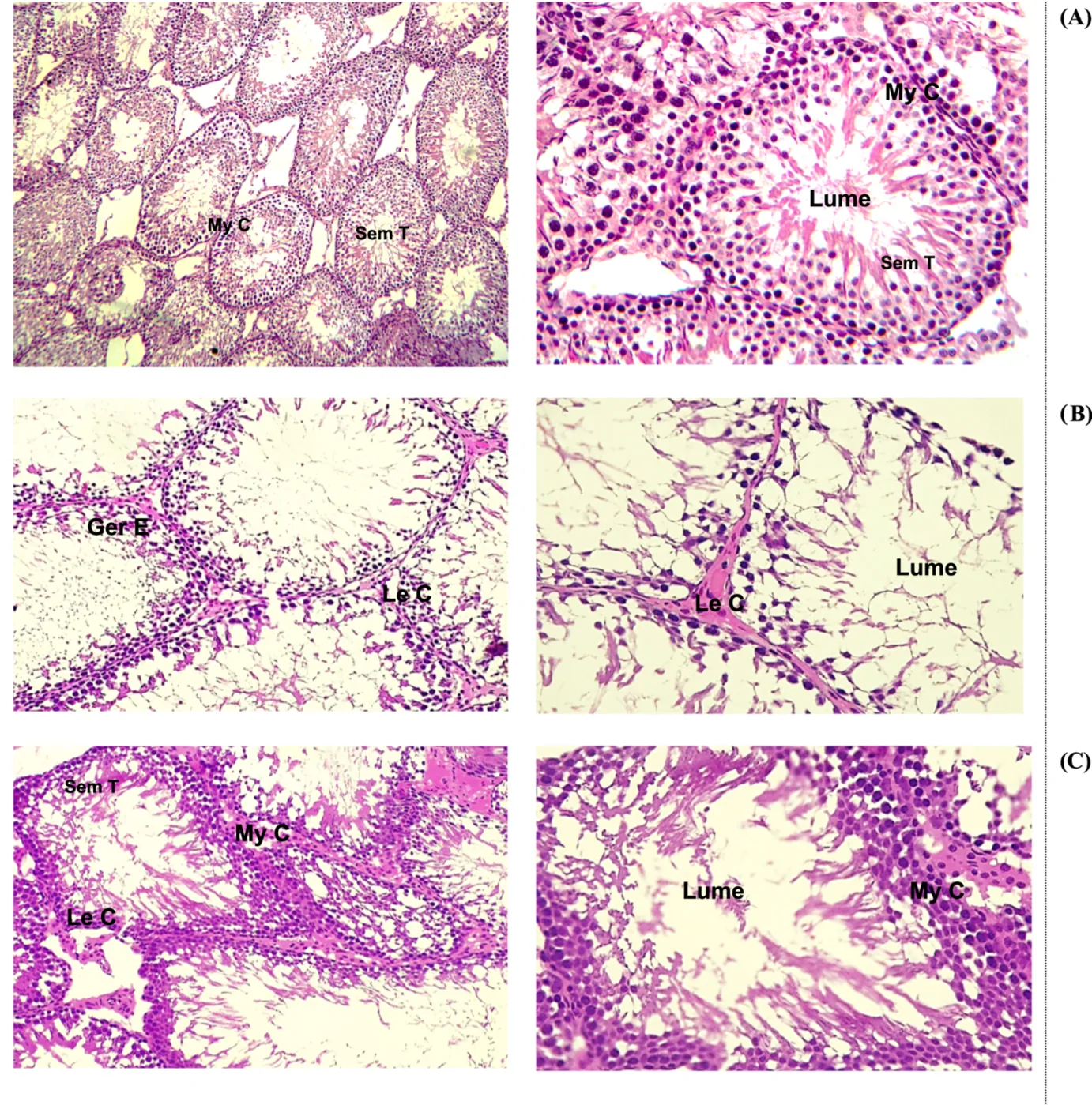
Protective effect of *Panax ginseng* on AlCl-nduced testis in the CG **(A)**, AlCl **(B)**, and AlCl/P-G **(C)**. Seminiferous tubule (Sem T), Leydig cells (Le C), Germinal Epithelium (Ger E), Myoid cells (My C). Images are captured by 10X and 40X magnifications.

### 3.4 SIRT1 protein expression

The data of NAD-dependent protein deacetylase sirtuin-1 (SIRT1) protein concentration in serum using enzyme-linked immunosorbent assay (ELISA; ng/mL) are shown in Table 6. There was a significant (*p* < 0.01) decrease in the AlCl₃ group compared to the CG. On the other hand, treatment with *Panax ginseng* in AlCl₃/P-G group showed a significant (*p* < 0.01) partial restoration of SIRT1 protein expression, with values remaining higher than those in the AlCl₃ and closer to the CG. Notably, the AlCl₃ /P-G showed absorption values like the CG, with no significant (*p* > 0.05) difference.

**Table 6:** NAD-dependent protein deacetylase SIRT1 protein expression in serum using enzyme-linked immunosorbent assay (ELISA; ng/mL) in CG, AlCl_3_, and AlCl_3_/P-G (*n* = 15/group, mean ± SEM).

Parameter	CG	AlCl₃	AlCl₃/P-G	SEM	Sig
SIRT1 Concentration (ng/mL)	7.45^a^	2.59^c^	5.07^b^	0.73	<0.0003

^a, b, c^ = Means with different superscripts in the same row differ significantly. *p* ≤ 0.01.

### 3.5 SIRT1 gene expression

The study evaluated SIRT1 mRNA expression in liver tissue homogenates via quantitative real-time PCR, normalizing results of GAPDH as housekeeping gene. Liver tissue was collected for molecular analysis because it is significantly affected by aluminum toxicity and has elevated levels of SIRT1. There was a significant difference in SIRT1 gene expression were noted among experimental groups (*p* < 0.01; Table 7). Aluminum exposure in the AlCl₃ group caused a 0.50-fold reduction in SIRT1 gene expression compared to the CG group. In contrast, co-administration of *Panax ginseng* in AlCl₃/P-G group significantly improved hepatic SIRT1 expression to 0.70-fold of CG levels, highlighting a partial protective effect against aluminum-induced suppression. The ^2^-ΔΔ^ Ct method showed that the AlCl₃/P-G group significantly increased SIRT1 mRNA levels compared to the AlCl₃ group (*p* < 0.01). Furthermore, qPCR and agarose gel electrophoresis results indicated that SIRT1 mRNA abundance was significantly lower in the AlCl₃ group compared to the control (CG) group, as shown by reduced band intensity (Figure 4). Conversely, the AlCl₃/P-G group exhibited increased band density, nearing that of the CG group, suggesting partial restoration of hepatic SIRT1 gene expression following *Panax ginseng* treatment.

**Table 7:** Relative quantitative real-time PCR (qPCR) of SIRT1 gene expression in liver tissue in CG, AlCl_3_, and AlCl_3_/P-G (n = 15 /group, mean ± SEM).

Parameter	CG	AlCl₃	AlCl₃/*P*-G	SEM	Sig
SIRT1 expression	1.00^a^	0.50^c^	0.70^b^	0.07	<0.0002

^a, b, c^ = Means with different superscripts in the same row differ significantly. *p* ≤ 0.01.

**Figure 4:**
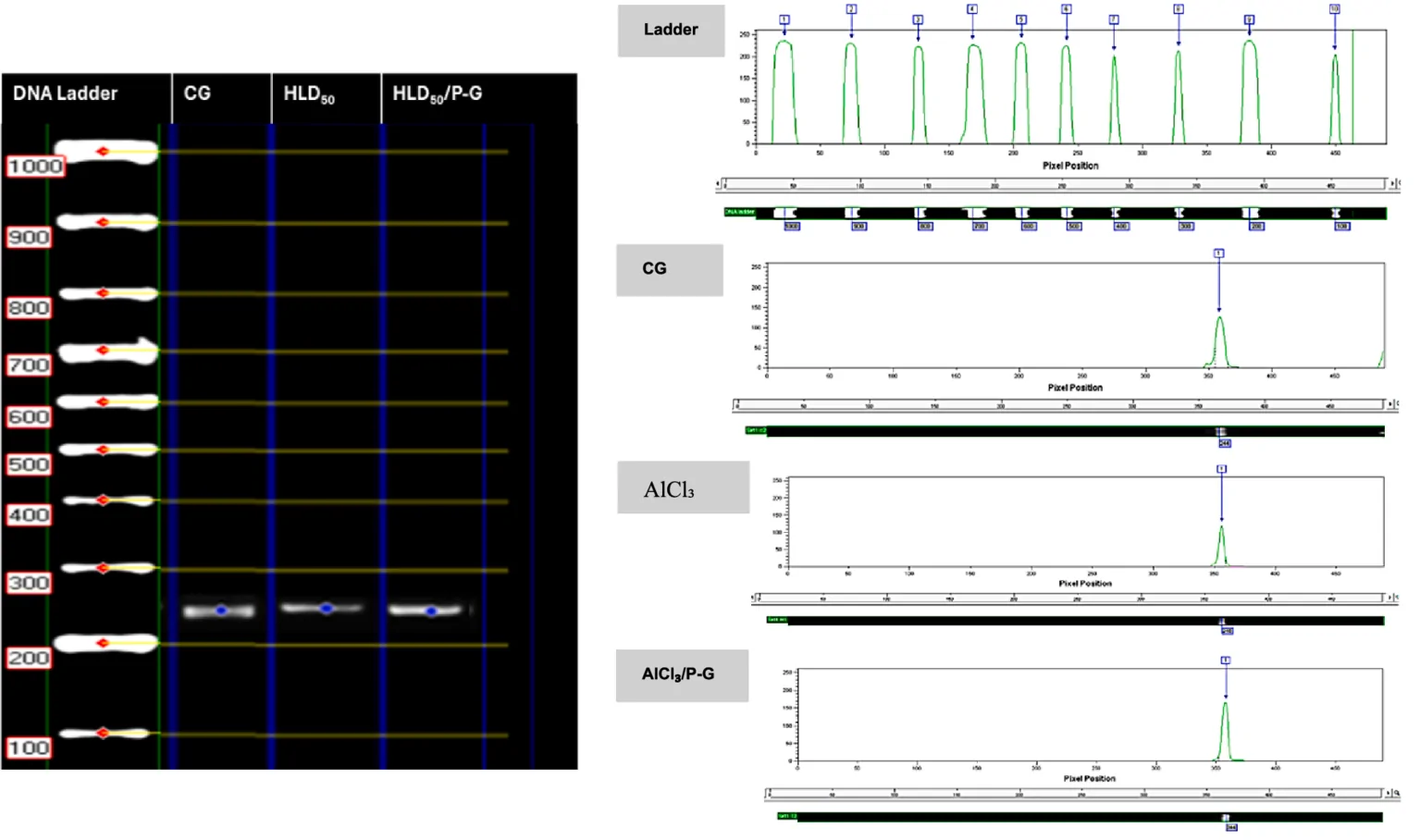
SIRT1 agarose gel electrophoresis showing SIRT1 PCR products from CG (lane 1), AlCl_3_ (lane 2), and AlCl_3_/P-G (lane 3), with DNA ladder.

## 4 Discussion

It has been confirmed that exposure to AlCl₃ leads to tissue damage and inflammatory dysfunction, necessitating research into effective protective interventions (31). This study investigated the protective potential of *Panax ginseng* against aluminum toxicity in albino rats, through biochemical and histopathological parameters of liver, kidney, and testicular function, and molecular modulation of SIRT1 signaling pathways given its pivotal role in cellular stress resistance and metabolic regulation, making it a potential target for mitigating toxin-induced tissue damage (32, 33). In both the AlCl₃ and AlCl₃/P-G groups, there was a statistically significant decrease in body weight compared to the CG at 7 and 14 days. The decrease in body weight in aluminum-exposed rats is attributed to reduced appetite and metabolism due to inflammation (34). In contrast, there was a significant improvement in body weight in the AlCl₃/P-G group at days 21 and 28 of treatment compared to the AlCl₃ and CG groups. These data suggest that *Panax ginseng* treatment improves body weight restoration by regulating adipocyte’s function and enhances appetite through leptin signaling and energy metabolism (35). Moreover, the AlCl₃/P-G group showed a significant improvement in total protein compared to AlCl₃ in several studies, *Panax ginseng* treatment has been shown to improve total protein and albumin in chemically induced liver injury models (36), and this improvement is attributed to reducing liver cell damage and restoring its functional structure (37). The lipid profile analysis indicated that the AlCl₃/P-G group had significantly lower values than the AlCl₃ group, while showing comparable values to the CG. The unexpected increase in HDL levels in the AlCl₃ group might result from a compensatory mechanism due to changes in lipid metabolism or be influenced by the high AlCl₃ dosage used. This effect may be attributed to *Panax ginseng*’s capacity to scavenge free radicals and block pancreatic lipase, which reduces fat absorption and synthesis (38). *Panax ginseng* also prevents fatty liver and hypertriglyceridemia in high-fat diet models (39). Furthermore, treatment with *Panax ginseng* in the AlCl₃/P-G group showed a significant improvement in creatinine, urea, ALT, and AST compared to AlCl₃. *Panax ginseng* treatment causes considerable reductions in serum urea and creatinine levels in toxin-exposed rats, indicating enhanced kidney function (40). These effects are linked to *Panax ginseng* antioxidant and anti-inflammatory characteristics, which help reduce renal oxidative damage and restore normal kidney function parameters (41). Notable improvements in serum ALT and AST levels confirmed hepatoprotection in AlCl₃/P-G compared to AlCl₃. In the liver, previous studies have shown that ginsenosides Rg1 and Rb1 can activated SIRT1 and promote Nrf2 activity (22); In the presented study, ginseng Co-treatment was association with higher hepatic SIRT1 mRNA expression, which may contribute to reduced hepatocellular injury. For renal protection, the improvement in kidney function parameters in the AlCl₃/*P-G* may be related SIRT1 upregulation as suggested by previous literature (22); although SIRT1 expression was measured only in the liver in this study. Ginsenosides can enhance spermatogenesis by controlling redox proteins and increasing the expression of related molecules (42). Overall, *Panax ginseng* exhibited significant protective effects across liver, kidney, and testicular tissues through SIRT1-mediated mechanisms. The data of SIRT1 protein expression showed a significant decrease in the AlCl₃ group compared to the CG. Consistent with our findings, previous studies have reported that aluminum exposure in rats decreases SIRT1 protein levels in various tissues, compromising the antioxidant system, worsening oxidative stress (32). Aluminum (AlCl₃ group) exposure caused a 0.50-fold reduction in SIRT1 gene expression compared to the CG group. Aluminum exposure induces oxidative stress by generating reactive oxygen species (ROS), which inhibits SIRT1 expression and activity (43). Consequently, this process results in heightened lipid peroxidation, inflammation, and apoptosis across hepatic, renal, and testicular tissues (44). In contrast, co-administration of *Panax ginseng* in AlCl₃/P-G group improved SIRT1 expression to 0.70-fold of CG levels. Computational predictions indicate that ginsenosides may interact with the NAD + binding domain of SIRT1, potentially enhancing its activation through hydrogen bonds, leading to increased fatty acid β-oxidation and improved cellular metabolism (45). Ginsenosides have been shown to enhance the expression and activity of SIRT1, offering protection against oxidative stress and inflammation (21, 22).

## 5 Study limitations

While the study highlights the protective potential of *Panax ginseng* against AlCl₃-induced toxicity in male albino rats, it has a methodological limitations. Notably, the absence of a ginseng-only group may limits the interpretation of its independent effects at the tested dosage of 250 mg/kg of body weight. Although the three-groups design (CG, AlCl₃, and AlCl₃/P-G) effectively demonstrated the protective potential of *Panax ginseng* against AlCl₃-induced multi-organ toxicity, the inclusion of a ginseng-only group would have provided definitive confirmation of the specific protective action of the extract and enabled a more comprehensive safety evaluation. Nevertheless, the marked differences observed between the AlCl₃, and AlCl₃/P-G groups compared to the DCG group, coupled with the integrated biochemical, histopathological, and molecular analyses, strongly suggest that the observed protective effects are attributable to *Panax ginseng*. Future research should include additional *Panax ginseng* groups to further validate the specific effects of *Panax ginseng*. Although SIRT1 concentration and mRNA alone do not confirm enzymatic activation, the increase in SIRT1 expression aligns with known effects of its activation, such as enhanced antioxidant defense and reduced inflammation, which is linked to significant improvements in biochemical markers of organ function and histopathological changes. This consistency with established mechanisms supports the notion that the SIRT1 antioxidant pathway mediates the protective effects of *Panax ginseng* against multi-organ toxicity induced by AlCl₃. Future studies should not only measure SIRT1 expression but also assess its deacetylase enzymatic activity to clarify the molecular mechanisms by which *Panax ginseng* protects against AlCl₃-induced toxicity.

## 6 Conclusion

The findings of this study highlight the potential protective role of *Panax ginseng* in AlCl₃-induced toxicity in albino rats. The results demonstrated its ability to partially mitigate structural and functional damage to liver, kidney, and testicular tissues, as well as to partially restore SIRT1 protein and gene expression. While these findings support the potential of *Panax ginseng* in protecting against aluminum-induced oxidative stress and tissue damage, further research with a ginseng-only control group is needed to confirm its independent protective effects.

## Data Availability

The raw data supporting the conclusions of this article will be made available by the authors, without undue reservation.
